# A Phase Ib/II Study of WNT974 + Encorafenib + Cetuximab in Patients With BRAF *^V600E^*-Mutant *KRAS* Wild-Type Metastatic Colorectal Cancer

**DOI:** 10.1093/oncolo/oyad007

**Published:** 2023-02-23

**Authors:** Josep Tabernero, Eric Van Cutsem, Elena Garralda, David Tai, Filippo De Braud, Ravit Geva, Mark T J van Bussel, Katia Fiorella Dotti, Elena Elez, María J de Miguel, Kevin Litwiler, Danielle Murphy, Michelle Edwards, Van Karlyle Morris

**Affiliations:** Vall d’Hebron Hospital Campus, Vall d’Hebron Institute of Oncology (VHIO), UVic-UCC, IOB-Quiron, Barcelona, Spain; University Hospitals Gasthuisberg Leuven and KU Leuven, Leuven, Belgium; START Madrid, Hospital Universitario HM Sanchinarro, Madrid, Spain; Division of Medical Oncology, National Cancer Centre Singapore, Singapore; Fondazione IRCCS Istituto Nazionale dei Tumori, Milan, Italy; Tel-Aviv Sourasky Medical Center, Tel-Aviv, Israel; Department of Clinical Pharmacology, The Netherlands Cancer Institute, Amsterdam, The Netherlands; Fondazione IRCCS Istituto Nazionale dei Tumori, Milan, Italy; Vall d’Hebron Barcelona Hospital Campus, Vall d’Hebron Institute of Oncology (VHIO), Universitat Autònoma de Barcelona, Barcelona, Spain; START Madrid, Hospital Universitario HM Sanchinarro, Madrid, Spain; Pfizer, Boulder, CO, USA; Pfizer, La Jolla, CA, USA; Pfizer, New York, NY, USA; Department of Gastrointestinal Medical Oncology, The University of Texas MD Anderson Cancer Center, Houston, TX, USA

**Keywords:** WNT974, encorafenib, cetuximab, metastatic, colorectal cancer

## Abstract

**Background:**

WNT974 is a small molecule inhibitor of Wnt signaling that specifically inhibits porcupine O-acyltransferase. This phase Ib dose-­escalation study evaluated the maximum tolerated dose of WNT974 in combination with encorafenib and cetuximab in patients with BRAF ^* V600E*^-mutant metastatic colorectal cancer with *RNF43* mutations or *RSPO* fusions.

**Patients and Methods:**

Patients received once-daily encorafenib and weekly cetuximab, in addition to once-daily WNT974, in sequential dosing cohorts. In the first cohort, patients received 10-mg WNT974 (COMBO10), which was reduced in subsequent cohorts to 7.5-mg (COMBO7.5) or 5-mg (COMBO5) after dose–limiting toxicities (DLTs) were observed. Primary endpoints were incidence of DLTs and exposure to WNT974 and encorafenib. Secondary endpoints were anti-tumor activity and safety.

**Results:**

Twenty patients were enrolled (COMBO10, *n* = 4; COMBO7.5, *n* = 6; COMBO5, *n* = 10). DLTs were observed in 4 patients, including grade 3 hypercalcemia (COMBO10, *n* = 1; COMBO7.5, *n* = 1), grade 2 dysgeusia (COMBO10, *n* = 1), and lipase increased (COMBO10, *n* = 1). A high incidence of bone toxicities (*n* = 9) was reported, including rib fracture, spinal compression fracture, pathological fracture, foot fracture, hip fracture, and lumbar vertebral fracture. Serious adverse events were reported in 15 patients, most frequently bone fracture, hypercalcemia, and pleural effusion. The overall response rate was 10% and disease control rate 85%; most patients achieved stable disease as their best response.

**Conclusion:**

Concerns surrounding the safety and lack of preliminary evidence of improved anti-tumor activity of WNT974 + encorafenib + cetuximab, compared with previous encorafenib + cetuximab data, ultimately led to study discontinuation. Phase II was not initiated.

**Trial registration:**

ClinicalTrials.gov, NCT02278133

**Implications for Practice**

This phase Ib dose-escalation study evaluates the maximum tolerated dose of WNT974, a Wnt inhibitor, when combined with encorafenib + cetuximab in patients with BRAF ^*V600E*^-mutant metastatic colorectal cancer with *RNF43* mutations or *RSPO* fusions. The addition of WNT974 to encorafenib + cetuximab was limited by severe toxicities. This study provides important information for future studies that include the addition of a Wnt inhibitor for the treatment of BRAF ^*V600E*^-mutant metastatic colorectal cancer.

## Introduction

Colorectal cancer (CRC) is the third most common cancer worldwide and the second most common cause of cancer deaths.^1^ The Wnt signaling pathway is altered in most CRCs, most commonly due to inactivating adenomatous polyposis coli (*APC)* mutations.^2-4^ In addition, some CRCs carry mutations in other genes, including *RNF43* mutations and *RSPO* fusions, which also activate the Wnt pathway and are mutually exclusive to *APC* mutations.^3,5-7^*RNF43* mutations are also associated with high microsatellite instability.^8^ These mutations serve as potential therapeutic targets for upstream inhibition of the Wnt pathway.^9^

*RNF43* and *RSPO* alterations in CRCs are associated with concurrent activating mutations in *BRAF*, a potent modulator of MAPK signaling.^4^ Approximately 10% of patients with metastatic CRC (mCRC) have a *BRAF* mutation, most commonly at the V600 codon.^10,11^ Patients with *BRAF*^*V600E*^-mutant mCRC typically have a worse prognosis than those with mCRC without *BRAF* mutations.^12^ BRAF inhibitor monotherapy shows little clinical benefit in patients with BRAF ^V600E^-mutant mCRC due to EGFR-mediated reactivation of the MAPK pathway in response to BRAF inhibition.^13-15^ Combining the EGFR inhibitor cetuximab with the BRAF inhibitor encorafenib has been proven successful in overcoming this feedback loop and improving survival outcomes in patients with BRAF ^*V600E*^-mutant mCRC.^16^

Crosstalk between the MAPK and Wnt signaling pathways suggests that they may act together to drive progression of CRC.^17^ Combining agents that target the aberrant signaling through both pathways may potentially improve outcomes in patients with CRC.^18^ WNT974 (formerly LGK974) is a small-molecule inhibitor of Wnt signaling, specifically inhibiting porcupine O-acyltransferase (*PORCN*).^19^ In vitro and in vivo, WNT974 demonstrated anti-tumor activity in CRC preclinical models.^20-22^ A phase I study of WNT974 reported a manageable safety profile in patients with advanced solid tumors, with the most frequently reported adverse events (AEs) across all doses being dysgeusia (50%), decreased appetite (45.7%), nausea (33.0%), fatigue (31.9%), and vomiting (30.9%).^23^

Here we report safety, pharmacokinetics (PK), and efficacy results from a phase Ib study to evaluate the combination of WNT974 + encorafenib + cetuximab in patients with BRAF ^*V600E*^-mutant mCRC with *RNF43* mutations or *RSPO* fusions.

## Methods

### Study Design and Patients

This open-label, multicenter, phase Ib dose-escalation study aimed to estimate the maximum tolerated dose (MTD) and/or recommended phase II dose (RP2D) of WNT974 + encorafenib + cetuximab in patients with BRAF ^*V600E*^-mutant mCRC harboring upstream Wnt pathway mutations. A single-arm phase II part of this study was planned to estimate the preliminary anti-tumor activity of the RP2D. The study protocol, informed consent form, and printed patient information materials were reviewed and approved by the independent ethics committee and/or local institutional review board for each site before any study procedures were performed (Supplemental Table S1). This study was conducted according to International Council for Harmonisation of Technical Requirements for Registration of Pharmaceuticals for Human Use guidelines concerning Good Clinical Practice, the Declaration of Helsinki, the European Union Clinical Trials Directive (2001/20/EC), Title 21 of the United States Code of Federal Regulations, and the practices and regulations of each participating nation. Written informed consent to participate in the study was obtained from each patient before any study-specific procedures were performed.

Eligible patients were 18 years or older; had mCRC with documented *KRAS* wild-type (WT) status, BRAF ^*V600E*^ mutation, and *RNF43* mutation and/or *RSPO* fusions detected by molecular testing; experienced disease progression after one or more prior standard-of-care regimens for mCRC; and had an Eastern Cooperative Oncology Group (ECOG) performance status ≤2 and evidence of measurable disease per Response Evaluation Criteria in Solid Tumors (RECIST) 1.1. A tumor biopsy (primary or metastatic, archival or newly obtained) was required at baseline. Patients with symptomatic brain metastases, symptomatic or untreated leptomeningeal disease, known acute or chronic pancreatitis, clinically significant cardiac disease, impaired hepatic function, or gastrointestinal disease or impaired gastrointestinal function that could significantly alter the absorption of WNT974 or encorafenib, as well as those receiving treatment with medication or consuming foods that were strong inhibitors/inducers of cytochrome CYP3A4/5, were excluded. Patients with prior treatment, including RAF inhibitors, Wnt pathway inhibitors, cetuximab, panitumumab, and/or other EGFR inhibitors were excluded from the phase II study only.

### Study Treatment and Procedures

Patients were enrolled sequentially into cohorts, and treatments were administered in 28-day cycles. At least 15 patients were planned for enrollment and 20 patients were enrolled in the phase Ib study. In all cohorts, 200 mg of encorafenib was administered orally once daily and cetuximab was administered intravenously once weekly starting with 400 mg/m^2^ in week 1 and 250 mg/m^2^ per week thereafter. In the first cohort, patients received 10 mg of oral WNT975 once daily (COMBO10). However, after 2 of the 4 patients in this cohort experienced dose-limiting toxicities (DLTs), the second cohort was initiated with a dose of 5 mg of WNT974 once daily (COMBO5). No patients in the second cohort reported a DLT. Therefore, cohort 3 was initiated with a dose of 7.5 mg of WNT974 once daily (COMBO7.5), and patient reported a DLT. Once the COMBO7.5 cohort was completed, an enrichment cohort was re-opened at the COMBO5 dose. Patients were treated until they experienced unacceptable toxicity that precluded any further treatment, disease progression, or treatment was discontinued at the investigator’s discretion or the patient’s request.

Patients who developed hypercalcemia (any grade) were treated with bisphosphonates per institutional standards. Due to emerging bone toxicity, patients were monitored by DEXA scan at screening, cycle 3 day 1, every 16 weeks, and as clinically indicated (following protocol amendment); patients who demonstrated bone loss either discontinued WNT974 treatment (in the case of bone fractures or new DEXA *T*-score of less than −2.5) or were treated with bisphosphonates and were monitored by a DEXA scan every 16 weeks.

### Endpoints

The primary endpoints of the phase Ib study were the incidence of DLTs and exposure to WNT974 and encorafenib as measured by PK parameters. A DLT was defined as an AE or abnormal laboratory value assessed as unrelated to disease, disease progression, intercurrent illness, or concomitant medications that occurred within the first 28 days of treatment with WNT974 + encorafenib + cetuximab. The secondary endpoints for this study included the objective response rate (ORR; RECIST 1.1), disease control rate (DCR), and frequency and severity of AEs (Common Terminology Criteria for Adverse Events v4.03).

Tumor assessments were performed at baseline (screening) and every 6 weeks after starting study treatment until disease progression, initiation of subsequent antineoplastic therapy, or death, whichever occurred first. End-of-treatment tumor assessment was conducted within 14 days of receiving the last dose or treatment discontinuation. Safety was assessed throughout the study and included physical examinations, vital signs, weight, performance status evaluation, laboratory evaluations, dermatologic evaluations, ophthalmological examinations, bone loss assessments, cardiac assessments, and documentation of all non-serious and serious AEs (SAEs).

### Pharmacokinetics

To assess plasma concentrations of WNT974, its active metabolite LHA333, and encorafenib, serial blood samples were collected on days 1 and 15 of cycle 1, and pre-dose blood samples were collected on days 2, 8, 16, and 22 of cycle 1, and on day 1 of cycles 2, 3, and 4. PK parameters were determined using noncompartmental methods. The PK analysis set included all patients with at least one available valid PK concentration measurement who received at least one dose of study drug.

### Statistical Analysis

Approximately 15 patients were planned to be enrolled in the phase Ib dose-escalation part of the study for the model to have reasonable operating characteristics relating to its MTD recommendation. The full analysis set (FAS) comprised all patients who received at least one full or partial dose of the assigned combination of drugs. The safety analysis set included all patients from the FAS who had at least one valid post-baseline safety assessment. The dose-­determining set consisted of all patients in the safety analysis set who either met the minimum exposure criterion (met if the patient received at least 21 of the 28 planned daily oral doses of WNT974 and encorafenib in the first 28 days of dosing, at least 50% [14 out of 28 days] of the planned combination doses of WNT974 and encorafenib administered together, and must have received the planned cetuximab loading dose and 2 additional planned cetuximab doses during the first 28 days of the study) and had sufficient safety evaluations (patients who did not experience a DLT during the first cycle if they had been observed for ≥28 days following the first dose and were considered by the Sponsor and Investigators to have enough safety data to conclude that a DLT did not occur) or who experienced a DLT during cycle 1. ORRs and DCRs were summarized using 95% CIs based on the exact binomial distribution.

## Results

### Patient and Disease Characteristics

Between March 2015 and April 2016, 20 patients were enrolled (data cutoff: August 25, 2017): 4 patients in COMBO10, 6 patients in COMBO7.5, and 10 patients in COMBO5. Patient and disease characteristics at baseline are shown in Table 1. Median patient age was 61 years and median number of previous treatment regimens was 2 (range: 1-6). Most patients were Caucasian (90%) and had an ECOG performance status of 0 (45%) or 1 (50%). Patients discontinued treatment due to disease progression (*n* = 15 [75%]), AEs (*n* = 3 [15%]), death (*n* = 1 [5%]), or physician decision (*n* = 1 [5%]) (Table 2). The median (range) duration of exposure to WNT974 + encorafenib + cetuximab for all patients was 22 weeks (0-80 weeks [Table 2]). The median (range) relative dose intensities for WNT974 and encorafenib were 86.3% (60%-100%) and 86.0% (60%-100%), respectively, for all patients. Because enrollment was discontinued and the phase II study was not initiated, MTD and RP2D were not determined.

**Table 1.** Patient and disease characteristics at baseline.

	COMBO5 (*n* = 10)	COMBO7.5 (*n* = 6)	COMBO10 (*n* = 4)	All patients (*N* = 20)
Median age, years (range)	63.5 (50-70)	60.5 (51-75)	60.5 (60-61)	61.0 (50-75)
Female sex	6 (60.0)	4 (66.7)	1 (25.0)	11 (55.0)
Race				
Caucasian	9 (90.0)	6 (100.0)	3 (75.0)	18 (90.0)
Asian	1 (10.0)	0	1 (25.0)	2 (10.0)
ECOG performance status				
0	3 (30.0)	4 (66.7)	2 (50.0)	9 (45.0)
1	6 (60.0)	2 (33.3)	2 (50.0)	10 (50.0)
2	1 (10.0)	0	0	1 (5.0)
Prior surgery	8 (80.0)	6 (100.0)	4 (100.0)	18 (90.0)
Number of prior regimens, median (range)	2.5 (1-6)	2.0 (2-3)	3.0 (2-6)	2.0 (1-6)
Primary site of cancer				
Colon	10 (100.0)	6 (100.0)	3 (75.0)	19 (95.0)
Rectum	0	0	1 (25.0)	1 (5.0)
Stage at initial diagnosis				
IIB	1 (10.0)	0	0	1 (5.0)
III	1 (10.0)	1 (16.7)	0	2 (10.0)
IIIB	1 (10.0)	1 (16.7)	0	2 (10.0)
IIIC	1 (10.0)	3 (50.0)	1 (25.0)	5 (25.0)
IV	4 (40.0)	1 (16.7)	1 (25.0)	6 (30.0)
IVA	1 (10.0)	0	0	1 (5.0)
IVB	1 (10.0)	0	1 (25.0)	2 (10.0)
Unknown	0	0	1 (25.0)	1 (5.0)
Stage at study entry				
IV	5 (50.0)	3 (50.0)	3 (75.0)	11 (55.0)
IVA	0	1 (16.7)	0	2 (10.0)
IVB	5 (50.0)	2 (33.3)	1 (25.0)	8 (40.0)
Time from initial diagnosis of primary site to start of study treatment (months)				
Mean (SD)	32.22 (27.1)	27.16 (15.3)	27.19 (14.5)	29.70 (21.2)
Median (min-max)	25.49 (3.3-92.0)	23.01 (14.9-55.4)	26.53 (12.2-43.5)	24.97 (3.3-92.0)
Time from initial diagnosis of primary site to first recurrence/progression (months)				
Mean (SD)	15.13 (13.7)	9.63 (5.0)	16.16 (16.3)	13.68 (12.0)
Median (min-max)	13.14 (2.3-49.6)	11.78 (0.0-13.2)	11.53 (1.9-39.7)	11.86 (0.0-49.6)
Time from most recent relapse/progression to start of study treatment (months)				
Mean (SD)	3.19 (3.3)	9.06 (16.9)	2.06 (0.9)	4.73 (9.4)
Median (min-max)	2.00 (0.9-12.0)	2.45 (0.9-43.4)	1.91 (1.1-3.3)	2.05 (0.9-43.4)
Types of lesions at baseline				
Target only	0	0	1 (25.0)	1 (5.0)
Both target and non-target	10 (100.0)	6 (100.0)	3 (75.0)	19 (95.0)

Data are *n* (%) unless indicated otherwise.

Abbreviation: ECOG, Eastern Cooperative Oncology Group.

**Table 2.** Treatment discontinuation and duration of exposure.

	COMBO5 (*n* = 10)	COMBO7.5 (*n* = 6)	COMBO10 (*n* = 4)	All patients (*N* = 20)
Treatment discontinued, *n* (%)	10 (100)	6 (100)	4 (100)	20 (100)
Primary reason for treatment discontinuation, *n* (%)				
Adverse event	1 (10.0)	1 (16.7)	1 (25.0)	3 (15.0)
Death	0	0	1 (25.0)	1 (5.0)
Physician decision	1 (10.0)	0	0	1 (5.0)
Progressive disease	8 (80.0)	5 (83.3)	2 (50.0)	15 (75.0)
Duration of exposure, weeks^a^				
Mean (SD)	29 (21)	21 (12)	26 (16)	26 (17)
Median (min, max)	25 (11, 80)	21 (0, 35)	22 (14, 48)	22 (0, 80)

^a^Duration of exposure was defined as (date of last exposure to study treatment - date of first administration of study treatment +1)/7.

Abbreviations: max, maximum; min, minimum.

### Dose-Limiting Toxicities

Three DLTs were reported: 1 patient in COMBO10 had grade 2 dysgeusia, 1 patient in COMBO10 had grade 4 increased lipase, and 1 patient in COMBO7.5 had grade 3 hypercalcemia (Table 3). All DLTs resolved following dose adjustment or temporary dose interruption.

**Table 3.** Dose-limiting toxicities by preferred term.

	COMBO5 (*n*= 10)	COMBO7.5 (*n* = 6)	COMBO10 (*n* = 4)	All patients (*N* = 20)
Any preferred term, *n* (%)		1 (16.7)	2 (50.0)	3 (20.0)
Increased alanine aminotransferase	^a^	0	0	1 (5.0)
Increased aspartate aminotransferase	^a^	0	0	1 (5.0)
Increased lipase	0	0	1 (25.0)	1 (5.0)
Hypercalcemia	0	1 (16.7)	0	1 (5.0)
Dysgeusia	0	0	1 (25.0)	1 (5.0)

^a^One patient with events of increased alanine aminotransferase and aspartate aminotransferase was originally included as having DLT events; upon investigation, the investigator categorized both events as not suspected to be study treatment-related and it was deemed they were misclassified as DLTs.

Abbreviations: DLT, dose-limiting toxicity.

### Safety

The most common AEs (any grade) were hypercalcemia, arthralgia, fatigue, and anemia (Table 4). Grade 3 or 4 AEs occurred in 16 patients, of whom ≥20% experienced hypercalcemia, hypophosphatemia, and increased aspartate aminotransferase. Treatment-related bone fractures occurred in 9 patients; 1 of these patients had metastatic bone lesions. SAEs occurred in 15 patients; the most common (occurring in ≥10% of patients) were bone fractures, hypercalcemia, and pleural effusion. One patient had serious bone pain. Four patients discontinued treatment due to an AE: bone fracture (*n* = 2), abdominal infection (*n* = 1), and infusion-related reaction (*n* = 1). Two patients died during the study (ie, within 30 days of the last treatment dose) due to disease progression.

**Table 4.** AEs occurring in ≥20% of all patients.

	COMBO5 (*n* = 10)		COMBO7.5 (*n* = 6)		COMBO10 (*n* = 4)		All patients (*N* = 20)	
	All grades	Grade 3/4	All grades	Grade 3/4	All grades	Grade 3/4	All grades	Grade 3/4
Any AE	10 (100.0)	7 (70.0)	6 (100.0)	5 (83.3)	4 (100.0)	4 (100.0)	20 (100.0)	16 (80.0)
Any SAE	8 (80.0)	6 (60.0)	4 (66.7)	3 (50.0)	3 (75.0)	3 (75.0)	15 (75.0)	12 (60.0)
Hypercalcemia	5 (50.0)	1 (10.0)	5 (83.3)	2 (33.3)	4 (100.0)	4 (100.0)	14 (70.0)	7 (35.0)
Arthralgia	5 (50.0)	0	4 (66.7)	0	4 (100.0)	0	13 (65.0)	0
Fatigue	6 (60.0)	0	2 (33.3)	0	3 (75.0)	0	11 (55.0)	0
Anemia	5 (50.0)	1 (10.0)	2 (33.3)	0	3 (75.0)	2 (50.0)	10 (50.0)	3 (15.0)
Bone fracture	4 (40.0)	1 (10.0)	4 (66.7)	3 (50.0)	1 (25.0)	1 (25.0)	9 (45.0)	5 (25.0)
Constipation	5 (50.0)	0	2 (33.3)	0	2 (50.0)	0	9 (45.0)	0
Nausea	4 (40.0)	0	4 (66.7)	0	1 (25.0)	0	9 (45.0)	0
Back pain	3 (30.0)	1 (10.0)	3 (50.0)	1 (16.7)	2 (50.0)	0	8 (40.0)	2 (10.0)
Hypophosphatemia	4 (40.0)	3 (30.0)	2 (33.3)	1 (16.7)	2 (50.0)	1 (25.0)	8 (40.0)	5 (25.0)
Pyrexia	5 (50.0)	0	2 (33.3)	0	1 (25.0)	0	8 (40.0)	0
Vomiting	4 (40.0)	0	2 (33.3)	0	2 (50.0)	0	8 (40.0)	0
Decreased appetite	3 (30.0)	0	2 (33.3)	1 (16.7)	2 (50.0)	0	7 (35.0)	1 (5.0)
Dysgeusia	2 (20.0)	0	2 (33.3)	0	3 (75.0)	0	7 (35.0)	0
Hypokalemia	3 (30.0)	0	2 (33.3)	0	2 (50.0)	1 (25.0)	7 (35.0)	1 (5.0)
Hypomagnesemia	3 (30.0)	0	1 (16.7)	0	3 (75.0)	1 (25.0)	7 (35.0)	1 (5.0)
Myalgia	3 (30.0)	0	2 (33.3)	0	2 (50.0)	0	7 (35.0)	0
Peripheral edema	3 (30.0)	0	0	0	4 (100.0)	0	7 (35.0)	0
Abdominal pain	2 (20.0)	0	2 (33.3)	0	2 (50.0)	0	6 (30.0)	0
Diarrhea	3 (30.0)	0	1 (16.7)	0	1 (25.0)	0	5 (25.0)	0
Hypocalcemia	1 (10.0)	0	1 (16.7)	0	3 (75.0)	1 (25.0)	5 (25.0)	1 (5.0)
Osteoporosis	2 (20.0)	0	2 (33.3)	0	1 (25.0)	0	5 (25.0)	0
Pain in extremity	2 (20.0)	0	1 (16.7)	0	2 (50.0)	1 (25.0)	5 (25.0)	1 (5.0)
Weight decreased	1 (10.0)	0	2 (33.3)	0	2 (50.0)	0	5 (25.0)	0
Increased aspartate aminotransferase	2 (20.0)	2 (20.0)	1 (16.7)	1 (16.7)	1 (25.0)	1 (25.0)	4 (20.0)	4 (20.0)
Increased blood bilirubin	1 (10.0)	0	2 (33.3)	1 (16.7)	1 (25.0)	1 (25.0)	4 (20.0)	2 (10.0)
Increased blood creatinine	0	0	1 (16.7)	0	3 (75.0)	1 (25.0)	4 (20.0)	1 (5.0)
Chills	2 (20.0)	0	1 (16.7)	0	1 (25.0)	0	4 (20.0)	0
Dyspnea	2 (20.0)	0	2 (33.3)	1 (16.7)	0	0	4 (20.0)	1 (5.0)
Headache	2 (20.0)	1 (10.0)	2 (33.3)	0	0	0	4 (20.0)	1 (5.0)
Infusion-related reaction	1 (10.0)	0	2 (33.3)	1 (16.7)	1 (25.0)	0	4 (20.0)	1 (5.0)
Musculoskeletal pain	4 (40.0)	0	0	0	0	0	4 (20.0)	0
Peripheral sensory neuropathy	1 (10.0)	0	2 (33.3)	0	1 (25.0)	0	4 (20.0)	0

Data are *n* (%).

Abbreviations: AE, adverse event; SAE, serious adverse event.

A phase II study was planned to assess the clinical efficacy and further safety of the treatment combination. However, enrollment was halted in April 2016 prior to initiation of phase II due to the emergence of 6 reported serious AEs of bone fracture across multiple studies involving WNT974. The preliminary efficacy data of the triplet combination (WNT974 + encorafenib + cetuximab) demonstrated limited improvement in clinical activity relative to the efficacy observed with doublet treatment (encorafenib + cetuximab) in patients with *BRAF* V600E-mutant mCRC in a separate ongoing clinical trial.^24^ This, in combination with the safety profile observed with this triplet combination (WNT974 + encorafenib + cetuximab), suggested that more restrictive patient selection, additional on-treatment monitoring, and prophylactic treatment to prevent bone resorption may be required, which would have hindered further development of the combination. Therefore, the decision was made to discontinue enrollment and to not initiate phase II of this study.

### Pharmacokinetics

Table 5 shows PK data for WNT974, its active metabolite LHA333, and encorafenib on day 15 of cycle 1. The mean areas under the plasma concentration-time curve (AUCs) of WNT974 and LHA333 increased in a dose-proportional manner. Median time to maximum concentration (*T*_max_) for WNT974, LHA333, and encorafenib ranged from 1 to 4 h across all treatment combinations. Mean half-life (*t*_1/2_) values for WNT974 were similar between the COMBO5 and COMBO7.5 cohorts (4.7 and 5.4 h, respectively) and were higher for the COMBO10 cohort (11.0 h). Similar trends occurred with the mean *t*_1/2_ of LHA333 (8.0, 7.4, and 15.0 h for COMBO5, COMBO7.5, and COMBO10, respectively). For encorafenib, mean AUC and maximum plasma drug concentration were generally comparable across all treatment combinations. Median *T*_max_ was observed at approximately 2 h for all cohorts, and mean *t*_1/2_ was similar across increasing doses of WNT974 at 4.3 h on day 15.

**Table 5.** Steady-state pharmacokinetics (day 15 of cycle 1) of WNT974, its active metabolite LHA333, and encorafenib.

	WNT974			LHA333			Encorafenib
	COMBO5 (*n* = 9)	COMBO7.5 (*n* = 5)	COMBO10 (*n* = 3)	COMBO5 (*n* = 9)	COMBO7.5 (*n* = 5)	COMBO10 (*n* = 3)	All cohorts (*N* = 17)
AUC_0−t_, h**·**ng/mL	220 (86.4)	384 (109)	460 (451)	117 (105)	241 (63.6)	239 (206)	13,700 (7130)
AUC_Τ_, h**·**ng/mL	227 (83.1)	384 (109)	542 (101)	141 (96.7)^†^	241 (63.7)	291 (101)^a^	13,800 (4940)^b^
*C*_max_, ng/mL	51.0 (23.2)	93.4 (55.7)	42.9 (37.1)	13.3 (7.48)	25.5 (8.03)	14.0 (12.9)	2840 (1430)
*T*_max_, h	1.00 (0.48-5.53)	1.02 (0.47-3.00)	4.00 (1.00-7.17)	1.98 (0.92-6.50)	1.95 (0.47-4.00)	4.00 (2.00-7.17)	1.95 (0.48-7.17)
*t*_1/2_, h	4.74 (2.17)	5.37 (0.697)	11.0 (9.12)^a^	7.95 (2.53)^c^	7.43 (1.07)	15.0 (12.5)^a^	4.29 (2.42)^b^

Data are mean (SD) for AUC_0−t_, AUC_*Τ*_, *C*_max_, and *t*_1/2_ and median (range) for *T*_max_.

^a^ *n* = 2; ^b^*n* = 16; ^c^*n* = 8.

Abbreviations: AUC_0−t_, area under the plasma concentration-time curve from time zero to infinity; AUC_*Τ*_, area under the plasma concentration-time curve during a dosing interval; *C*_max_, maximum plasma drug concentration; *t*_1/2_, terminal half-life; *T*_max_, time to *C*_max_.

### Anti-Tumor Activity

Overall, the confirmed ORR and DCR were 10% (95% CI, 1.2-31.7) and 85% (95% CI, 62.1-96.8), respectively. Most patients (*n* = 15, 75%) achieved stable disease as their best response to treatment. Two patients (both on the COMBO5 dose) had a partial response. No complete responses were observed. A summary of response outcomes is shown in Table 6. There were 18 patients evaluable for *RNF43* mutation status, 8 of whom had *RNF43* mutant. There did not appear to be any correlation between the best percentage change from baseline in sum of longest diameters and *RNF43* mutation status (based on local testing; data not shown).

**Table 6.** Response outcomes.

	COMBO5 (*n* = 10)	COMBO7.5 (*n* = 6)	COMBO10 (*n* = 4)	All patients (*N* = 20)
Best overall response				
Complete response	0	0	0	0
Partial response	2 (20.0)	0	0	2 (10.0)
Stable disease	7 (70.0)	4 (66.7)	4 (100.0)	15 (75.0)
Progressive disease	1 (10.0)	0	0	1 (5.0)
Unknown	0	2 (33.3)	0	2 (10.0)
Overall response rate	2 (20.0)	0	0	2 (10.0)
95% CI	2.5-55.6	—	—	1.2-31.7
Disease control rate	9 (90.0)	4 (66.7)	4 (100.0)	17 (85.0)
95% CI	55.5-99.7	22.3-95.7	39.8-100.0	62.1-96.8

Data are *n* (%) unless indicated otherwise.

## Discussion

This phase Ib trial of the triplet combination of WNT974 + encorafenib + cetuximab showed the safety of the combination was not established in patients with BRAF ^*V600E*^-mutant *KRAS* WT mCRC harboring Wnt pathway mutations. Taken together, these data suggest further development of the triplet combination would require additional on-treatment monitoring to address safety concerns, including prophylactic treatments to prevent bone resorption, potentially hindering further development of the combination. Hence, enrollment was discontinued and after a safety and efficacy evaluation, following the consensus agreement of the study investigators, the phase II study was not initiated.

In this single-arm trial of encorafenib + cetuximab + WNT974 in the same type of patient population, bone-related complications were among the most common and the most serious treatment-related toxicities observed. The combination treatment was associated with a higher incidence of bone-related toxicities, including increased susceptibility to fractures, bone pain, and hypercalcemia, compared with doublet treatment (encorafenib + cetuximab) or WNT974 monotherapy. In the phase III BEACON trial for BRAF ^*V600E*^-mutant mCRC, there were no reports of osteopenia/osteoporosis, hypercalcemia, or bone fracture (of any grade) among the 218 patients treated with encorafenib + cetuximab or in patients treated with encorafenib + binimetinib + cetuximab.^25^ In another phase Ib trial evaluating encorafenib + cetuximab with or without alpelisib, hypophosphatemia was very common in the encorafenib + cetuximab arm, with 19% of patients experiencing grade 3/4.^26^ In a previous phase I trial of WNT974, 6 patients experienced 7 bone-related toxicities, of which 5 were considered related to the study treatment. Suspected treatment-related bone-associated toxicities included, osteoporosis, pathological fracture, osteopenia, and spinal fracture.^23^ Hypophosphatemia can be a cause of bone toxicity also due to decreased bone mineralization (prolonged hypophospatemia over months to years leads to rickets and osteomalacia), and this may be what was contributing to the additive effect on bone toxicity observed, even if on their own encorafenib and cetuximab did not lead to clinically detectable bone fractures.

The role of the Wnt pathway in bone homeostasis, repair, and regeneration is well known.^27^ A study in mice showed that bisphosphonates can mitigate acute bone loss due to PORCN inhibition.^28^ However, in clinical practice, phase I trials of Wnt pathway inhibitors in which patients received prophylactic bisphosphonates have shown mixed results; in patients with advanced solid tumors, ipafricept (a Wnt pathway inhibitor) was well tolerated and alterations in bone remodeling were mostly reversible with bisphosphonate therapy. Alternatively, trials of vantictumab (an anti-Frizzled antibody) + chemotherapy for the treatment of patients with HER2-negative breast cancer or pancreatic cancer closed early due to high rates of bone fracture.^29-31^

The PK data for WNT974 and LHA333 should be interpreted with caution. Ninety-one of 446 WNT974 and LHA333 samples included in the calculation of the WNT974 and LHA333 PK parameters were analyzed after the validated storage stability time of 345 days. None of the samples analyzed for encorafenib were affected by stability issues. The study was not adequately powered to assess specific ­biomarker-related hypotheses. However, the molecular landscape should be evaluated in this patient population in future trials to better understand how best to treat these patients.

The 2 patients who achieved a response had a duration of response of 106 and 291 days, respectively. Neither patient had any specific characteristics that appeared to enhance their chance of response. The patient with the longest duration of response experienced an SAE of grade 2 hypercalcemia, which resolved, followed by a grade 3 AE that was not suspected to be treatment-related but resulted in permanent discontinuation of the study treatment.

It is unknown whether *RSPO* fusions contributed to differential responses. It has been shown that *RSPO* fusions occur less frequently compared with *RNF43* mutations but Wnt expression levels were not evaluated.^7^ Wnt expression levels were not evaluated, and its role as a true oncogenic driver is not yet fully understood.^32^

## Conclusion

Although the triplet combination in this study was limited by toxicities, the rationale for combining encorafenib and cetuximab with a Wnt pathway inhibitor holds promise. Future clinical trials involving Wnt pathway inhibitors must mitigate the risk of bone-related toxicities by intensively monitoring bone homeostasis and including bone protective measurements in their clinical trial protocols and patient selection. While novel combination approaches beyond inhibition of BRAF and EGFR are warranted for patients with BRAF ^*V600E*^-mutant mCRC, it appears that the addition of a PORCN inhibitor to this combination resulted in excessive toxicity that did not justify further study.

## Supplementary Material

oyad007_suppl_Supplementary_Material

## Data Availability

The data underlying this article are available in the article and in its online supplementary material. Upon request, and subject to review, Pfizer will provide the data supporting the findings of this study. Subject to certain criteria, conditions, and exceptions, Pfizer may also provide access to the related individual de-identified participant data. See https://www.pfizer.com/science/clinical-trials/trial-data-and-results for more information.
