# Supplementary material for: A Phase Ib/II Study of WNT974 + Encorafenib + Cetuximab in Patients With BRAF V600E-Mutant KRAS Wild-Type Metastatic Colorectal Cancer
Source: Oncologist. 2023 Feb 23;28(3):230–8. doi: 10.1093/oncolo/oyad007 (PMC10020809; doi:10.1093/oncolo/oyad007)
Supplement: oyad007_suppl_Supplementary_Material [file oyad007_suppl_supplementary_material.docx]

**Supplemental Table 1**. List of Independent Ethics Committees/Institutional Review Boards

| **Sites that Received Study Drug and Consented a Subject** | | |
| --- | --- | --- |
| **Site Number** | **Principal Investigator** | **Independent Ethics Committee/Institutional Review Board** |
| Australia | | |
| 1001 | Desai | Melbourne Health Human Research Ethics Committee  Grattan Street, Human Research Ethics Committee  Directorate, Post Office  6 East, Main Building  Parkville, Victoria 3050  Australia |
| Belgium | | |
| 6501 | Van Cutsem | Commissie voor Medische Ethiek - ZNA Middelheim  Lindendreef 1, AZ Middelheim  Antwerpen 2020  Belgium |
| Canada | | |
| 7501 | Kennecke | UBC BC Cancer Agency Research Ethics Board  2410 Lee Avenue  Victoria, British Columbia  V8R 6V5  Canada |
| 7502 | Sawyer | Health Research Ethics Board of Alberta Cancer  Committee  1500 10104 103 Avenue NW  Edmonton, Alberta  T5J 4A7  Canada |
| 7503 | Siu | University Health Network Research Ethics Board  700 Bay Street  17th Floor, Suite 1700  Toronto, Ontario  M5G 1Z6  Canada |
| Israel | | |
| 4501 | Geva | Tel Aviv Sourasky EC  6 Weitzman Street  Tel-Aviv 64239  Israel |
| Italy | | |
| 7001 | De Braud | Comitato Etico Fondazione IRCCS Istituto Nazionale  dei Tumori di Milano  Via Venezian 1  Milano, Lombardia 20133  Italy |
| Netherlands | | |
| 6001 | Schellens | Raad van Bestuur Nederlands Kanker Instituut Antoni  Van Leeuwenhoek  Plesmanlaan 121  Amsterdam, Noord-Holland  1066 CX  Netherlands |
| Singapore | | |
| 3001 | Tai | Singhealth Centralised Institutional Review Board  7 Hospital Drive, Singhealth Office Of Research  Blk A, #03-01, Singhealth Research Facilities  Singapore 169611  Singapore |
| Spain | | |
| 8501 | Tabernero | CEIC Hospital Universitari Vall d'Hebron  Passeig de la Vall d'Hebron, 119-129  Institut de Recerca  Edificio Hospital Materno-Infantil  Planta 13a  Barcelona 8035  Spain |
| 8502 | Garralda | CEIC Grupo Hospital de Madrid  Avenida Monteprincipe, 25  Secretaria del Comite Etico de Investigacion Clinica  Grupo Hospitales de Madrid  Boadilla del Monte  Madrid 28660  Spain |
| United States | | |
| 5001 | Britten | Institutional Review Board for Human Research  Office of Research Integrity  19 Hagwood Ave, Suite 601, MSC 857  Charleston, SC 29425  United States |
| 5003 | Morris | University of Texas MD Anderson Cancer Center  Institutional Review Board  7007 Bertner Ave, Unit 1637  Houston, TX 77030  United States |
| 5005 | Yaeger | Memorial Sloan Kettering Cancer Center Institutional  Review Board/Privacy Board  1275 York Avenue  New York, NY 10065  United States |
